# Validity of a low-cost Lichtenstein open inguinal hernia repair simulation model for surgical training

**DOI:** 10.1007/s10029-019-02093-6

**Published:** 2019-12-02

**Authors:** T. Nazari, M. P. Simons, M. H. Zeb, J. J. G. van Merriënboer, J. F. Lange, T. Wiggers, D. R. Farley

**Affiliations:** 1 Department of Surgery, Erasmus University Medical Center, Rotterdam, The Netherlands; 2 Department of Surgery, OLVG, Amsterdam, The Netherlands; 3 Department of Surgery, Mayo Clinic, Rochester, MN USA; 4 Department of Educational Development and Research, Faculty of Health, Medicine and Life Sciences Maastricht University, Maastricht University, Maastricht, The Netherlands; 5 Incision Academy, Amsterdam, The Netherlands

**Keywords:** Surgical education, Simulation model, Liechtenstein, Open inguinal hernia repair

## Abstract

**Purpose:**

Simulation training allows trainees to gain experience in a safe environment. Computer simulation and animal models to practice a Lichtenstein open inguinal hernia repair (LOIHR) are available; however, a low-cost model is not. We constructed an inexpensive model using fabric, felt, and yarn that simulates the anatomy and hazards of the LOIHR. This study examined the fidelity, and perceived usefulness of our developed simulation model by surgical residents and expert surgeons.

**Methods:**

A total of 66 Dutch surgical residents and ten international expert surgeons were included. All participants viewed a video-demonstration of LOIHR on the simulation model and subsequently performed the surgery themselves on the model. Afterward, they assessed the model by rating 13 statements concerning its fidelity (six model, three equipment, and four psychological) and six usefulness statements on a five-point Likert scale. One-sample Wilcoxon signed-rank test was used to compare to the neutral value of 3.

**Results:**

The fidelity was assessed as being high by residents [model 4.00 (3.00–4.00), equipment 4.00 (3.00–4.00), psychological 4.00 (3.00–4.00); all *p*’s < 0.001] and by expert surgeons [model 4.00 (3.00–4.00), *p* = 0.025; equipment 4.00 (3.00–5.00), *p* < 0.001; psychological 4.00 (3.00–4.00), *p* = 0.053]. The usefulness was rated high by residents and experts, especially the usefulness for training of residents [residents 4.00 (4.00–5.00), *p* < 0.001; experts 4.50 (3.75–5.00), *p* = 0.015].

**Conclusion:**

Our developed Lichtenstein open inguinal hernia repair simulation model was assessed by surgical residents and expert surgeons as a model with high fidelity and high potential usefulness, especially for the training of surgical residents.

**Electronic supplementary material:**

The online version of this article (10.1007/s10029-019-02093-6) contains supplementary material, which is available to authorized users.

## Introduction

In current surgical education, learning by simulation training is a frequent adjunct to preparation for real operating room experiences [1]. Surgical simulation models allow the trainees to gain their experience in a safe environment [2], without risking patient safety [3].

One of the core procedures in training surgical residents is the inguinal hernia repair. The open inguinal hernia repair with the placement of a tension-free mesh was introduced in 1984 by Lichtenstein [4]. Even though the use of the laparo-endoscopic repair of the inguinal hernia is rising, the European Hernia Society’s updated guideline for the treatment of inguinal hernia in adult patients recommended both the Lichtenstein and the laparo-endoscopic technique as the best evidence-based options [5]. The open inguinal hernia repair technique is simpler to teach compared to the laparo-endoscopic techniques [6]. In many low-resource regions, laparoscopic surgery is not available.

Simulation models to practice the open inguinal hernia operation, such as a computer simulation [7–9] or animal models [10], are available. However, to our knowledge, no low-cost model simulating the Lichtenstein open inguinal hernia repair (LOIHR) has been published. We sought to construct a model using inexpensive materials that simulates the anatomical structures and hazards of the LOIHR.

Fidelity determines the extent to which the simulation model resemblances reality. It measures the degree in which the appearance and behavior of the simulation model match the real experience [11]. Fidelity consists of three domains suggested by Rehmann: ‘environment’ which was, in this case, the simulation model, ‘equipment’ and ‘psychological’ [12]. This study aimed to examine the fidelity, and potential usefulness of our developed Lichtenstein open inguinal hernia repair simulation model.

## Methods

### Participants and design

This study was conducted among surgical residents and expert surgeons in order to assess the open inguinal hernia repair simulation model. The surgical residents were invited for inclusion during the education days of the Dutch Association of Surgery. The surgical residents were shown a video-demonstration of the LOIHR with the placement of a tension-free mesh on the model first. Afterward, the surgical residents performed the surgery themselves on the model and filled out the rating scale questionnaires. Participation was anonymous and voluntary. This study does not require institutional board review according to Dutch law.

The international expert surgeons had significant experience in performing the LOIHR. Ten expert surgeons were invited per email for participation. All experts confirmed participation. After confirmation of participation, they were sent a package containing a LOIHR simulation model and an instruction letter including their login credentials to a website where they could view the video-demonstration of the LOIHR performed on the simulation model and where they could fill out the rating scales concerning the model. First, they were asked to view the video-demonstration, then to perform the surgery themselves, and last to fill out the rating scales. Informed consent was obtained from all individual participants included in the study.

### Lichtenstein open inguinal hernia repair simulation model

**Fig. 1:**
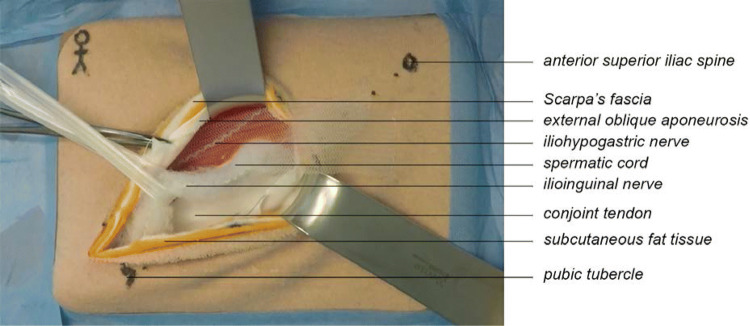
Open inguinal hernia simulation model, left male groin

The LOIHR simulation model mimicked the human male groin region, including the abdominal wall layers and the contents of the inguinal canal. Each structure included in the model was crucial for the LOIHR. Positioned within the correct layers were the hazardous structures, such as the superficial epigastric vessels, the spermatic cord, and the ilioinguinal, iliohypogastric nerves and genital branch of the genitofemoral nerve (Fig. 1).

The model was constructed using low-cost materials. The tan-colored fabric was used to mimic the skin, white felt to mimic Scarpa’s fascia, and yellow felt layers to mimic the subcutaneous fat, and red felt was used to mimic the internal oblique muscle. A broad white braided elastic band mimicked the conjoint tendon. White cotton layers were used to represent the anterior rectus sheath and the external oblique aponeurosis, including an opening to simulate the external ring of the inguinal canal and a fold representing the inguinal ligament. The spermatic cord was constructed using batting. Within this batting, a small transparent plastic bag was added to simulate an indirect hernia. Red, blue, yellow, and white yarn were used to mimic the arteries, veins, nerves, and the vas deferens, respectively. The material cost per model was less than five US dollars. This model was identical to the one used for the video-demonstration.

The model was first developed at the Mayo Clinic and was initially reported by Rowse et al. [13]. After using the initial model for training of surgical residents in the Mayo Clinic and Ghana and critical assessment of the model by the senior authors, adjustments were made to optimize the model. Due to the adjustments, the conjoint tendon and a separate anterior rectus sheath were added to the model. The spermatic cord was pasted to the conjoint tendon to allow trainees to dissect it. Finally, the iliohypogastric nerve was adjusted so it would run towards the subcutaneous fat tissue underlying the skin.

### Video demonstration

The video-demonstration showing the LOIHR on the simulation model lasted for 8:00 min (video-demonstration LOIHR available online). The surgery was based on the description of Amid [14] and was divided into steps using the step-by-step framework [15] (Appendix A). The step-by-step framework breaks down surgical procedures based on anatomical structures and implants, into steps and substeps.

### Rating scales

After the surgical residents and surgeons operated on the simulation model, they were instructed to fill out the rating scales. The questions were adapted from a previously used questionnaire in a study on fidelity and its different domains using 6 model, 3 equipment, and 4 psychological statements [16] (Appendix B). An example of a model-related statement was “This simulation model provides a realistic representation of the abdominal layers.” The equipment fidelity was assessed using statements as “On this simulation model, I could demonstrate the precise movements of the open inguinal hernia repair.” Statements as “My experience with the simulation model seemed (overall) consistent with my real-world experiences” were used to assess the psychological fidelity.

The usefulness of the model as a teaching entity and for specific groups (medical students, residents, surgeons) was assessed using six statements (Appendix C). All statements were rated on a five-point Likert scale (1 = Strongly disagree, 2 = Disagree, 3 = Neutral, 4 = Agree, 5 = Strongly agree).

### Statistical analysis

All statistical analyses were performed using SPSS (IBM Corp. Released 2016. IBM SPSS Statistics for Windows, Version 24.0. Armonk, NY: IBM Corp.). Descriptive data were presented as medians and interquartile range (IQR) of the statements per domain of fidelity and for the usefulness were calculated. The Mann Whitney *U* test was used to compare the surgical residents and expert surgeons. One-sample Wilcoxon signed-rank test was used to compare the median per domain of fidelity and usefulness to the neutral value of 3. The internal consistencies for the three domains within fidelity and for the usefulness rating scales were determined using Cronbach’s *α*. *p* values of less than 0.05 will be considered statistically significant.

## Results

In total, 66 Dutch surgical residents were included. Their average age was 32 years (Table 1). None of the surgical residents were in their first year of training: 3 in their second, 32 in their third, 24 in their fourth, and seven in their fifth or sixth year.

**Table 1:** Demographics surgical residents

	Surgical residents (*n* = 66)
Age (median; range)	32 (29–36)
Sex (%)	
Female	35.4%
Male	64.6%
Year surgical training (mean ± SD)	3.55 ± 0.778
Total amount of open inguinal hernia repairs seen (%)	
< 10	4.5%
10–20	12.1%
20–30	18.2%
> 30	65.2%
Total amount of endoscopic inguinal hernia repairs seen (%)	
< 10	10.6%
10–20	24.2%
20–30	21.2%
> 30	43.9%
Total amount of open inguinal hernia repairs performed (%)	
< 5	7.6%
5–10	6.1%
10–15	16.7%
> 15	69.7%
Total amount of endoscopic inguinal hernia repairs performed (%)	
< 5	45.5%
5–10	12.1%
10–15	6.1%
> 15	36.4%

The included experts were ten surgeons from seven different countries and three different continents. As can be seen in Table 2, the average age was 55 years (range 37–69). One expert had less than 10 years of post-residency experience, one had 10–20 years, and eight experts had more than 20 years. Five expert surgeons had performed more than 3000 open inguinal hernia repairs in total, and two expert surgeons had performed more than 10.000. Five experts had published more than 50 hernia-related papers.

**Table 2:** Demographics expert surgeons

	Expert surgeons (*n* = 10)
Age (median; range)	55 (37–69)
Sex (%)	
Female	2
Male	8
How many years of surgical experience (postgraduate) do you have? (*n*)	
< 10	1
10–20	1
> 20	8
What is the total amount of open inguinal hernia repairs performed in your clinic annually? (*n*)	
< 200	2
200–400	4
400–600	1
> 600	3
What is the total amount of open inguinal hernia repairs performed personally by you in a year? (*n*)	
< 100	4
100–200	5
> 300	1
What is the total amount of open inguinal hernia repairs performed personally by you in total? (*n*)	
> 100	1
> 1.000	3
> 3.000	2
> 6.000	1
> 10.000	2
Unknown	1
What is the total amount of endoscopic inguinal hernia repairs performed personally by you in a year? (*n*)	
< 100	8
100–200	1
> 300	1
How many hernia-related papers did you publish in total? (*n*)	
< 25	1
> 25	3
> 50	3
> 75	2
Unknown	1

As shown in Table 3, the model fidelity was rated 4.00 [3.00–4.00] by the surgical residents and the expert surgeons (*U* = 277.00, *p* = 0.393). The surgical residents rated the equipment fidelity 4.00 [3.00–4.00] compared to 4.00 [3.00–5.00] rated by the experts (*U* = 2796.5, *p* = 0.615). The psychological fidelity was found to be both 4.00 [3.00–4.00] by the surgical residents and the experts (*U* = 5054, *p* = 0.892). For the surgical residents, these were all significantly higher compared to the neutral value of 3 (all *p*’s < 0.001). In the case of the experts, this was true for model (*Z* = 2.24, *p* = 0.025) and equipment (*Z* = 3.20, *p* = 0.001). The internal consistency of the fidelity rating scale was found to be good (environment 0.876, equipment 0.836, psychological 0.857).

**Table 3:** Fidelity

	Surgical residents n = 66	Expert surgeons *n* = 10	Experts vs residents	Surgical residents vs neutral value of 3	Experts vs neutral value of 3
	Median [IQR]	Median [IQR]	*p* value^a^	*p* value^b^	*p* value^b^
Model Cronbach *α* = 0.876	4.00 [3.00 – 4.00]	4.00 [3.00 – 4.00]	0.044*	< 0.001*	0.025*
Equipment Cronbach *α* = 0.836	4.00 [3.00 – 4.00]	4.00 [3.00 – 5.00]	0.615	< 0.001*	0.001*
Psychological Cronbach *α* = 0.857	4.00 [3.00 – 4.00]	4.00 [3.00 – 4.00]	0.892	< 0.001*	0.053

IQR interquartile range [Q1–Q3]

^*^Statistically significant

^a^Analyzed using Mann Whitney *U* test

^b^Analyzed using one sample Wilcoxon signed rank test

The usefulness of the LOIHR simulation model was assessed to be 4.00 [3.00–5.00] by the surgical residents and the surgeon experts (Table 4, *U* = 11,759.5, *p* = 0.946, Cronbach *α* = 0.824). In both groups, this was significantly different compared to the neutral value of 3 (surgical residents *p* < 0.001; experts *p* < 0.001). Both groups found the model useful in teaching the importance of the open inguinal hernia repair and of placing a tension-free mesh. The surgical residents found the model to be useful for the training of surgical residents (Z = 6.48, p < 0.001) and for medical students (*Z* = 6.56, *p* < 0.001). The experts found it useful for training surgical residents (*Z* = 2.43, *p* = 0.015).

**Table 4:** Usefulness

	Surgical residents *n* = 66	Expert surgeons *n* = 10	Experts vs. residents	Surgical residents vs. neutral value of 3	Experts vs. neutral value of 3
	Median [IQR]	Median [IQR]	*p* value^a^	*p* value^b^	*p* value^b^
Usefulness of the LOIHR simulation model (Cronbach *α* = 0.824)	4.00 [3.00 – 5.00]	4.00 [3.00 – 5.00]	0.946	< 0.001*	< 0.001*
The LOIHR simulation model teaches the importance of performing the open inguinal hernia repair	4.00 [4.00 – 5.00]	4.00 [4.00 – 5.00]	0.717	< 0.001*	0.012*
The LOIHR simulation model teaches the importance of placing a tension-free mesh	4.00 [4.00 – 5.00]	4.50 [3.50 – 5.00]	0.414	< 0.001*	0.024*
The LOIHR simulation model is a useful tool to learn open inguinal hernia repair surgery	4.00 [4.00 – 5.00]	4.00 [3.75 – 5.00]	0.421	< 0.001*	0.018*
The LOIHR simulation model is useful for training of experts	3.00 [2.00 – 4.00]	3.50 [1.00 – 4.25]	0.838	0.590	0.903
The LOIHR simulation model is useful for training of surgical residents	4.00 [4.00 – 5.00]	4.50 [3.75 – 5.00]	0.817	< 0.001*	0.015*
The LOIHR simulation model is useful for training of medical students	4.00 [3.00 – 5.00]	3.50 [2.00 – 5.00]	0.487	< 0.001*	0.248

IQR interquartile range [Q1–Q3]

^*^Statistically significant

^a^Analyzed using Mann Whitney *U* test

^b^Analyzed using one sample Wilcoxon signed rank test

## Discussion

Simulation models to practice the open inguinal hernia model, such as a computer simulation [7, 8] or animal models [10] are available. However, to our knowledge no low-cost model simulating the open inguinal hernia repair is available. Surgical residents assessed our developed low-cost Lichtenstein open inguinal hernia repair simulation model as a model with high fidelity. The surgeon experts only valued the model to have a high equipment fidelity. Both the surgical residents and the experts rated the usefulness of the model as high, especially for the training of surgical residents.

Animal or cadaveric models are typical examples of high-fidelity models, and they may resemble reality more than our model [2]. In a comprehensive systematic review and meta-analysis, the authors found that a simulation model could be of high- or low fidelity depending on which domains were assessed [17, 18]. In contrary to the three domains of fidelity (model, equipment and psychological) we used during this study, Allen, et al. divided fidelity into two domains: physical fidelity (how the simulator appears) which may resemble our model domain, and functional fidelity (what the simulator does) which resembles our equipment domain [19]. Allen’s domains do not include the psychological domain. When this study would have only used the domains of Allen, the surgical residents and the experts would have both assessed the fidelity of the model as high. Apart from which domains need to be assessed, the learning objectives of a simulator are more relevant. The learning objectives should determine the degree of fidelity of a simulator [20]. In our simulation model, the aim of creating the model was to carefully position the hazardous structures within the model to achieve the highest resemblance to reality. The trainee could cause the same complications in our model as in a real patient.

Simulation models are a step between theoretical learning and performing surgery on patients, as it allows trainees to learn and practice without risking patient safety [3]. With this LOIHR simulation model, the anatomical and procedural knowledge, together with the surgical skills of trainees, could be assessed. These features made the model particularly useful for training surgical residents in an uncomplicated case. Both the surgical residents and the expert surgeons found this to be true. However, in many cases, the reality differs due to variations caused by the patient (e.g., obesity), the disease (e.g., direct hernia) and anatomy (e.g., abnormal position of the iliohypogastric nerve). These variations demand an adjustment of the surgical procedure. Our model lacked these variations consciously, as this simulation model allows the trainee safe repetition until proficient to be able to perform the standard surgery supervised on a patient. The trainee will encounter the numerous variations possible during the LOIHR when he or she performs the surgery supervised in the OR. The point of proficiency can be determined by systematically tracking the competence of the trainees, for example by using the essential step by step description of the surgical procedure and the Observational Clinical Human Reliability Assessment (OCHRA) [21]. The OCHRA assesses the errors made during the surgical procedure.

Advantages of the model are low-cost, producible by anyone, and usable anywhere and allows widespread usage, especially in low-resource environments. The LOIHR simulation model was constructed using non-expensive materials. The price of all components to construct one model was less than 5 US dollars. The cost of computer simulators, animal or cadaveric models often lacked in reports; however, in our own experience, these resources are significantly (> 100 times) more expensive than our model [2, 8, 22]. The construction of a single model took 30 min. With proper instruction, anyone can construct the model. We have experience with making the model by tailors in Ghana. We made an instruction video on how to make the model, and after the first initial trials, the model was very accurate. Last, in order to practice with this model, only basic surgical instruments are needed, in comparison to advanced computer systems, or an animal or cadaveric laboratory.

Concluding, our developed low-cost Lichtenstein open inguinal hernia repair simulation model was assessed as a model with high fidelity and high perceived usefulness, especially for the training of surgical residents.

## Electronic supplementary material

Below is the link to the electronic supplementary material.

Supplementary file1 (DOCX 107 kb)

Supplementary file2 (DOCX 82 kb)

Supplementary file3 (DOCX 41 kb)
