# Supplementary material for: Validity of a low-cost Lichtenstein open inguinal hernia repair simulation model for surgical training
Source: Hernia. 2019 Dec 2;24(4):895–901. doi: 10.1007/s10029-019-02093-6 (PMC7395906; doi:10.1007/s10029-019-02093-6)
Supplement: Supplementary file 1 — Supplementary file1 (DOCX 107 kb) [file 10029_2019_2093_MOESM1_ESM.docx]

Appendix A. Step-by-step description open inguinal hernia repair

| **Step** | **Substep (structure)** | **Action** | **Specification** |
| --- | --- | --- | --- |
| 1. External oblique aponeurosis exposure | Skin | Incise | Incise the skin for a length of approximately 5 cm in the line between anterior superior iliac spine to the pubic tubercle. |
|  | Subcutaneous tissue | Incise | Incise the subcutaneous tissue until Scarpa’s fascia is reached. |
|  |  |  | HAZARD Superficial epigastric vessels damage  During the incision of the subcutaneous tissue caution should be taken for the superficial epigastric vessels. |
|  | Superficial epigastric vessels | Transect | Transect the superficial epigastric vessels in order to prevent postoperative hemorrhage. |
|  | Scarpa's fascia | Incise | Incise Scarpa’s fascia to expose the fat tissue overlying the external oblique aponeurosis. |
|  | Subcutaneous tissue | Incise | Incise the subcutaneous tissue overlying the external oblique aponeurosis to expose it. |
| 2. Inguinal canal exposure | External oblique aponeurosis | Identify | Identify the external oblique aponeurosis as a white plane with oblique fiber lines. |
|  |  | Incise | Incise the aponeurosis of the external oblique muscle in the direction of the fibers. Extend the incision toward the external inguinal ring medially. |
|  |  |  | Hazard - The ilioinguinal nerve on top of the spermatic cord should be identified in order to prevent injuring it. |
|  |  | Dissect | Dissect the external oblique aponeurosis by developing the plane between the aponeurosis of the external oblique muscle and the internal oblique muscle caudally, while avoiding the ilioinguinal nerve. |
| 3. Spermatic cord mobilization | Spermatic cord | Isolate | Isolate the spermatic cord completely from the floor of the inguinal canal, the transverse fascia and the pubic bone. |
|  |  | Encircle | Encircle the spermatic cord with a penrose drain. |
| 4. Hernia sac removal | Hernia sac | Identify | Identify the hernia sac. |
|  |  | Remove | Remove the hernia sac by first rotating the hernia sac around its own axis. Then the hernia sac is transected after clamping and tying the rotated hernia sac. |
| 5. Mesh placement | Inguinal ligament | Expose | Expose the inguinal ligament which is the lower edge of the inguinal canal. |
|  | Mesh | Trim | Trim the mesh to fit the inguinal canal. |
|  |  | Fixate - medial | Fixate the mesh medially to the distal anterior rectus sheath with 2 cm overlap over the pubic bone. |
|  |  |  | HAZARD - Pubic periosteum damage  During medial fixation of the mesh to the anterior rectus sheath, care should be taken not to include the pubic periosteum in this suture as this may result in chronic pain. |
|  |  | Fixate - caudal | Fixate caudally by continuing with a running suture on the lower edge of the inguinal ligament until the level of the internal inguinal ring. Do not continue this suture beyond the lateral border of the internal inguinal ring. |
|  |  |  | HAZARD - Femoral vessels and nerve damage  During the caudal fixation, avoid damaging the femoral vessels and nerve which run just underneath the inguinal ligament. Damage is avoided by only taking small bites of the lower edge of the inguinal ligament (1-2 mm). |
|  |  | Trim | Trim the mesh from lateral to medial until the medial border of the internal inguinal ring, creating two tails. The superior tail is approximately 2/3 of the width of the mesh and the inferior tail is approximately 1/3 of the width of the mesh. |
|  |  | Position | Position the mesh under the spermatic cord, and subsequently maneuver the spermatic cord between the two tails. Then, pass the superior tail over the inferior tail to create a prosthetic internal inguinal ring. |
|  |  | Fixate | Fixate both tails with a single non-absorbable suture to the inguinal ligament. |
|  |  | Position | Position the tails of the mesh under the external oblique aponeurosis laterally and cranially. |
|  |  | Fixate - cranial | Fixate the superior margin of the mesh with one or two interrupted absorbable sutures to the aponeurosis of the internal oblique muscle, while avoiding the iliohypogastric nerve. |
|  |  |  | HAZARD - Iliohypogastric nerve damage  Avoid damage or entrapment of the iliohypogastric nerve, preferably by identification. Make a transverse suture rather than a longitudinal suture, as this minimizes the risk of nerve entrapment. If necessary a separate cut can be made into the mesh to free the iliohypogastric nerve. If the nerve cannot be freed from the mesh, transect this nerve and bury the iliohypogastric nerve in the oblique internal muscle. |
| 6. Wound closure | External oblique aponeurosis | Close | Close the aponeurosis of the external oblique muscle, creating a new external ring. |
|  | Scarpa's fascia | Close | Close Scarpa’s fascia. |
|  | Skin | Close | Close the skin. |
