# Supplementary material for: Validity of a low-cost Lichtenstein open inguinal hernia repair simulation model for surgical training
Source: Hernia. 2019 Dec 2;24(4):895–901. doi: 10.1007/s10029-019-02093-6 (PMC7395906; doi:10.1007/s10029-019-02093-6)
Supplement: Supplementary file 2 — Supplementary file2 (DOCX 82 kb) [file 10029_2019_2093_MOESM2_ESM.docx]

Appendix B. Fidelity rating scale

| **Model** | |
| --- | --- |
| This simulation model provides a realistic representation of the abdominal layers |  |
| This simulation model provides a realistic representation of inguinal canal |  |
| This simulation model provides a realistic representation of spermatic cord |  |
| This simulation model provides a realistic representation of the nerves |  |
| This simulation model provides a realistic representation of the hazards during the surgery |  |
| The general performance using this simulator was close in comparison to my general performance in the clinical settings |  |
| **Equipment** | |
| On this simulation model, I could demonstrate the precise movements of the open inguinal hernia repair |  |
| I could use all tools/equipment required to perform this procedure in a manner which is close in comparison to the real procedure (OR) |  |
| Fixating the mesh was accurate on this simulation model |  |
| **Psychological** | |
| While performing the procedure on the simulation model, it felt like I was doing the procedure on a patient |  |
| I felt comfortable performing the procedure |  |
| The feel of the equipment made me feel as if I were actually doing the real procedure (in OR) |  |
| My experience with the simulation model seemed (overall) consistent with my real-world experiences |  |
