# Supplementary material for: Validity of a low-cost Lichtenstein open inguinal hernia repair simulation model for surgical training
Source: Hernia. 2019 Dec 2;24(4):895–901. doi: 10.1007/s10029-019-02093-6 (PMC7395906; doi:10.1007/s10029-019-02093-6)
Supplement: Supplementary file 3 — Supplementary file3 (DOCX 41 kb) [file 10029_2019_2093_MOESM3_ESM.docx]

Appendix C. Usefulness rating scale

| The open inguinal hernia simulation felt model … | |
| --- | --- |
|  | … teaches the importance of performing the open inguinal hernia repair |
|  | … teaches the importance of placing a tension-free mesh |
|  | … is a useful tool to learn open inguinal hernia repair surgery |
|  | … is useful for training of experts |
|  | … is useful for training of surgical residents |
|  | … is useful for training of medical students |
